# Production of a high purity, C‐tagged hepatitis B surface antigen fusion protein VLP vaccine for malaria expressed in *Pichia pastoris* under cGMP conditions

**DOI:** 10.1002/bit.28181

**Published:** 2022-07-22

**Authors:** Ekta Mukhopadhyay, Florian Brod, Philip Angell‐Manning, Nicola Green, Richard D. Tarrant, Frank J. Detmers, Emma J. Bolam, Ioana N. Baleanu, Mark Hobson, Gary Whale, Susan J. Morris, Rebecca Ashfield, Sarah C. Gilbert, Jing Jin, Simon J. Draper, Sarah P. Moyle, Eleanor L. Berrie, Adrian V. S. Hill

**Affiliations:** ^1^ Clinical BioManufacturing Facility, The Jenner Institute, Nuffield Department of Medicine University of Oxford Oxford UK; ^2^ The Jenner Institute, Nuffield Department of Medicine University of Oxford Oxford UK; ^3^ Thermo Fisher Scientific Leiden The Netherlands

**Keywords:** cGMP, malaria, vaccine

## Abstract

Virus‐like particles (VLPs) induce strong humoral and cellular responses and have formed the basis of some currently licensed vaccines. Here, we present the method used for the production of R21, a VLP‐based anti‐sporozoite malaria vaccine, under current Clinical Good Manufacturing Practice regulations (cGMP). Previous preclinical studies in BALB/c mice showed that R21 produced almost complete protection against sporozoite challenge with transgenic *Plasmodium berghei* parasites. Here, we have modified the preclinical production process to enable the production of sufficient quantities of highly pure, clinical‐grade material for use in human clinical trials. The R21 construct was re‐engineered to include a C‐tag to allow affinity‐based separation from the major contaminant alcohol oxidase 1 (AOX 1, ~74 kDa). To our knowledge, this is the first use of C‐tag technology to purify a VLP vaccine candidate for use in human clinical trials. The R21 vaccine has shown high‐level efficacy in an African Phase IIb trial, and multiple clinical trials are underway to assess the safety and efficacy of the vaccine. Our findings support the future use of C‐tag platform technologies to enable cGMP‐compliant biomanufacturing of high purity yeast‐expressed VLP‐based vaccines for early phase clinical trials when clinical grade material is required in smaller quantities in a quick time frame.

## 1 INTRODUCTION

The burden of infectious diseases has greatly reduced over the years in part by the development and use of safe and effective vaccines. Technological advances in bioprocessing, such as the use of novel expression systems, new purification technologies, and single‐use systems, have made the manufacture of vaccines a cheaper, faster, and simpler process (Eric, 2013). While production of large volumes of vaccines at a low cost is an important goal in the development of new vaccines, early Phase 1 trials often require the production of smaller batches of high purity product in a short time frame. This has previously been achieved by using the C‐Tag affinity purification platform that makes use of a short C‐terminal tag, composed of the four amino acids EPEA (Jin et al., 2017, 2018). Virus‐like particles (VLPs) as vaccines have gained interest due to their particulate nature; capable of eliciting excellent humoral and also cellular immune responses (Boisgerault et al., 2002; Layton et al., 1996; Villa et al., 2006; Wagner et al., 1998). Examples of commercial native VLP‐based vaccines are GlaxoSmithKline's Engerix® (hepatitis B virus) and Cervarix® (human papillomavirus), and Merck and Co. Inc.'s Recombivax HB® (hepatitis B virus) and Gardasil® (human papillomavirus). These vaccines use insect cell or yeast platforms for the production of VLPs.

Malaria remains a global health threat with 219 million cases worldwide in 2017, and no significant progress recorded in reducing global malaria cases between 2015 and 2017 (WHO, 2018). Of the approximately 40 malaria vaccine candidates that have reached clinical trials in the last 15 years (Coffeng et al., 2017), RTS,S is the only vaccine to have completed Phase 3 testing. It reduced clinical incidence by 39% and severe malaria by 31.5% among children aged 5–17 months, entered a pilot implementation program (WHO, 2018) and has now been recommended by WHO to be used in childhood vaccination in a wider population (WHO, 2021). The limited success of previous malaria vaccine efforts might relate in part to the complex life cycle of the parasite. During its two‐host life cycle, in human and mosquito, *Plasmodium falciparum* undergoes numerous different morphological changes in five different host tissues (Arama & Troye‐Blomberg, 2014) and expresses ~5500 proteins (Cunningham et al., 2016). One of the most abundant proteins is the circumsporozoite protein (CSP), a 397 amino acid (Porter et al., 2013) surface antigen on the sporozoite of the malaria parasite (Coppi et al., 2005). In *P. falciparum*, the highly conserved central repeat region of the CSP protein mainly consists of multiple copies of the tetrapeptide NANP. This region is conserved between parasite variants and is the target of protective antibodies in rodent malaria models (Hill, 2006).

The RTS,S vaccine utilizes the hepatitis B surface antigen (HBsAg) VLP as a platform to display malaria epitopes on the surface of particles approximately 22 nm in size. The central repeat region and the C‐terminus of CSP have been fused to HBsAg to generate the RTS fusion protein and co‐expressed in *Saccharomyces cerevisiae* yeast with unmodified recombinant HBsAg (S). This spontaneously forms lipid‐protein particles upon cell lysis, resulting in hybrid particles formed from a mixture of the RTS and S proteins, at a ratio of 1:4. The CSP fusion protein thus comprises only 20% of the protein content in the particle (Gordon et al., 1995; Heppner et al., 2005; Regules et al., 2011) with the excess of S antigen being required to facilitate assembly of the VLP.

**Figure 1:**
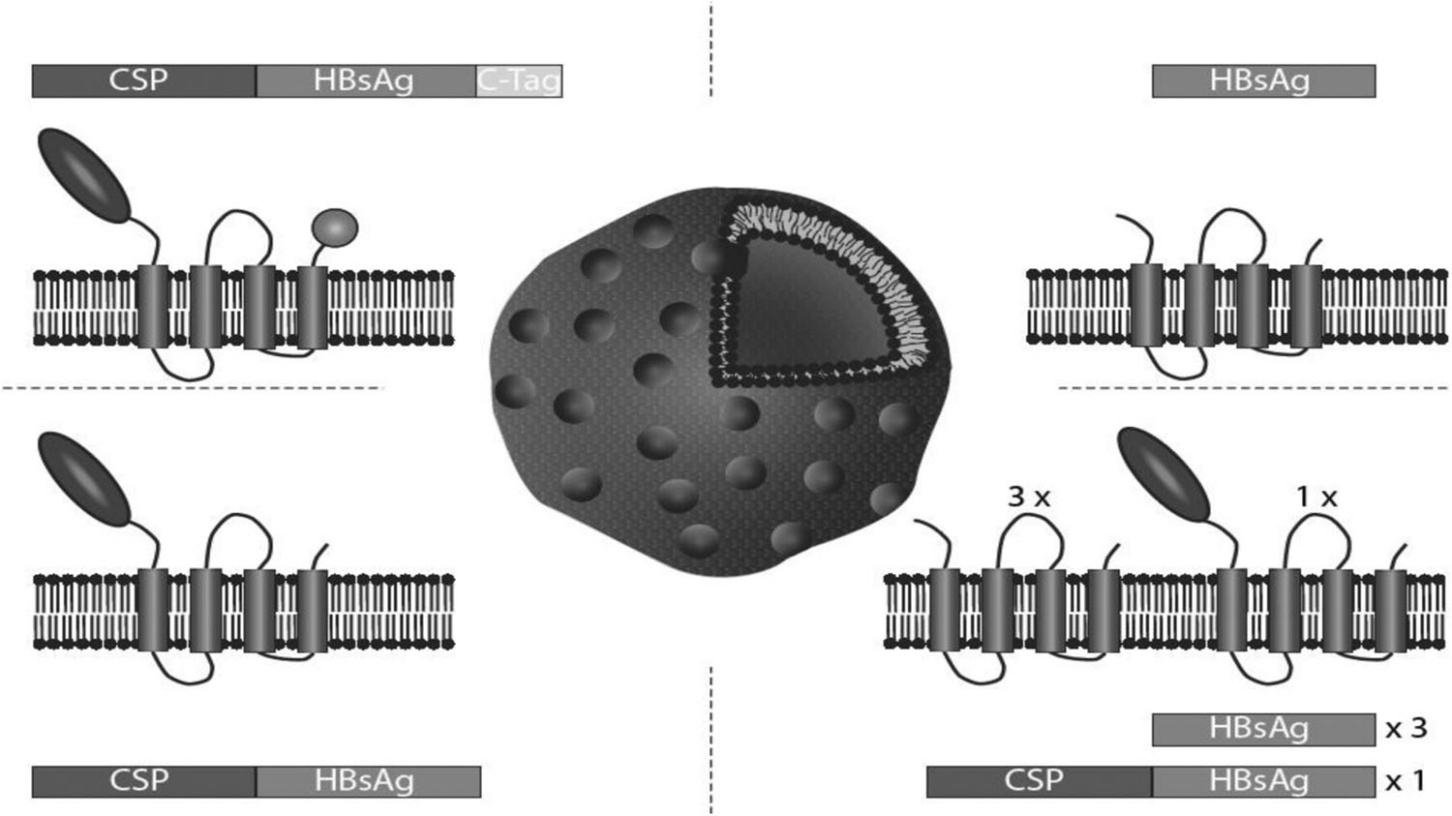
Difference between RTS,S and R21c. VLPs based on HBsAg (center) consist of a host cell‐derived lipid bilayer into which multiple copies of the quadruple‐membrane‐spanning HBsAg are embedded (top right). In RTS,S (bottom right), one in four HBsAg subunits is replaced with the CSP‐HBsAg fusion protein, while R21 (bottom left) consists solely of the membrane‐embedded fusion protein. R21c (top left) is similar to R21, but in addition to the N‐terminally fused CSP contains the four amino acids C‐Tag fused to the HBsAg C‐terminus. HBsAg, hepatitis B surface antigen; VLP, virus‐like particle.

R21 is a next‐generation particle‐based vaccine and has been developed at the Jenner Institute, University of Oxford in recent years (Collins et al., 2017). R21 particles are formed from a single CSP‐hepatitis B surface antigen (HBsAg) fusion protein, without free S monomers being included in the VLP. It consists of the C‐terminal half region of CSP from *P. falciparum* strain NF54, containing 19 copies of the NANP central repeat, fused to the N‐terminus of HBsAg. Although similar to RTS,S, R21 displays CSP at fourfold higher density on the VLP surface (Figure 1). In preclinical studies, R21 induced a protective immune response when used in combination with a variety of adjuvants, producing high levels of antibodies to the NANP repeat as well as a moderate T‐cell response in BALB/c mice. When administered with the adjuvant Matrix‐M™ (Novavax, Inc.), R21 induces almost 100% sterile protection in mice (Collins et al., 2017). Matrix‐M™ is a saponin‐based adjuvant, containing an extract from the *Quillaja saponaria* Molina tree, that can help induce high levels of long‐lasting antibodies (Bengtsson et al., 2016).

Preclinical production of R21 had limitations for the manufacture of sufficient quantities of clinical grade material following cGMP guidelines. In preparation for clinical trials, we have modified the purification process to align with production under cGMP conditions. The main focus of the development of the process was laid on specific purification steps to obtain high purity clinical grade material and upstream steps like different media types and cell culture systems like bioreactors were not explored.

## 2 MATERIALS AND METHODS

### 2.1 Materials

The starting material for the clinical batch was produced by transformation of *Pichia pastoris* (*Pichia pink* strain 1; Invitrogen, 2014) by electroporation with the linearized pPink‐HC‐R21‐EPEA plasmid and culturing on Pichia Adenine Dropout (PAD) medium. For clone selection, the transformed strain was taken through three rounds of cell cloning by re‐streaking on YPDA (1% Yeast extract [Sigma‐Aldrich #Y1625], 2% Peptone [Sigma‐Aldrich #18332], 2% bacteriological agar [Sigma‐Aldrich #A5306]) plates. For Master Cell Bank (MCB) production, the culture was expanded in YPD (1% Yeast extract, 2% Peptone) media. For expression check, expansion and production of the clinical batch, the cultures were expanded in BMGY (1% yeast extract, 2% peptone, 100 mM potassium phosphate, 1.34% Yeast Nitrogen Base, 0.0004% Biotin, 1% glycerol) media and induced using BMMY (1% yeast extract, 2% peptone, 100 mM potassium phosphate, 1.34% Yeast Nitrogen Base [Sigma‐Aldrich #Y0626], 0.0004% Biotin, 0.5% methanol) media. Pichia lysis buffer (PLB; 0.1% Triton, 1 mM EDTA, 1 mM MgCl_2_, 10 mM Tris) was used for lysis, clarification, and the Capto Core 700 chromatography step. The Capto Core 700 resin (GE Healthcare Lifesciences) was used for the removal of low molecular weight contaminants and R21c monomers and CaptureSelect™ C‐tag affinity resin (Jin et al., 2018) was used for selective binding of R21c VLPs containing a C‐tag. The C‐tag resin consisted of 13 kDa single chain camelid antibodies (NbSyn2) produced in yeast and immobilized onto aldehyde‐activated agarose beads 35 µm in size (De Genst et al., 2010). The antibodies selectively bind to a four amino acid residue peptide sequence (E‐P‐E‐A, glutamic acid‐proline‐glutamic acid‐alanine) fused to the C‐terminus of the protein. The resin has been developed by BAC B.V (now Thermo Fisher Scientific) in the Netherlands under cGMP conditions. No significant immune response to the C‐tag was observed in preclinical models (Jin et al., 2017). The Capto Core 700 resin column (ID 50 mm, 196 ml) and C‐tag resin column (ID 26 mm, 50 ml) was packed by BioToolomics Ltd. The major contaminant AOX 1 was eluted from the C‐tag affinity column using Elution buffer 1 (20 mM Tris, 0.1 M MgCl_2_, pH 7), followed by elution buffer 2 (20 mM Tris, 2 M MgCl_2_ buffer, pH 7) to elute R21c VLPs. For the final buffer exchange step, dialysis was performed using 30 mL slide‐a‐lyzer cassettes of 10 K MWCO (Thermo Fisher) in pre‐autoclaved low endotoxin 4 L bottles in formulation buffer (20 mM Tris, 15% sucrose (w/v), 30 mM MgCl_2_, pH 7.4). Before filling, the dialyzed material was filtered through 0.45 µm polyvinylidene fluoride (PVDF) (Millipak® Express 20) filter followed by filtration through 0.22 µm PVDF (Millipak® Express 20) filter and 0.3 ml was filled into sterile and depyrogenated type 1 glass vials.

#### 2.1.1 Equipment

Growth and expansion of cultures were performed using 250 or 1000 ml baffled flasks (Corning Inc.) in a Kuhner shaker incubator (Model #ISF1‐X). Lysis was performed by single pass of the suspension through a high‐pressure cell disruption system (Model: TS5/40/AE/LA; Constant Systems Limited) at 40 kpsi. Clarification of the lysate was achieved using a disposable C0HC depth filter (Millistak+® Pod 0.027 m^2^; Merck Millipore) and a milliflex pump. The filter incorporates multiple graded‐density layers and adsorptive, positively charged filter media enabling the removal of cellular debris, host cell proteins, and host cell DNA. The C0HC filter was autoclaved before use as per the manufacturer's instructions at >121°C, 60 min. To assess clone expression, postlysis, an XK16 column (GE Healthcare Life Sciences) packed with 5 ml of C‐tag affinity resin was used on the AKTA Prime Plus liquid chromatography system (GE Healthcare Life Sciences). Capto Core 700 chromatography and C‐tag affinity chromatography steps for the production of clinical batch using confirmed clones, were performed on the AKTA Purifier 100 (GE Healthcare Lifesciences). Final 0.22 µm PVDF (Millipak® Express 20) filtration and filling were performed in an isolator (class 100; Envair).

### 2.2 Methods

Cloning of R21c containing plasmid pPink‐HC‐R21‐EPEAR21c was expressed in *P. pastoris* (*Pichia pink* strain 1; Invitrogen, 2014) under an AOX 1 promoter. The R21c protein sequence (see Supporting Information: Material 1) contains from N‐ to C‐terminus: the NANP repeats (×19) and the C‐terminus of CSP (*P. falciparum*) followed in frame by HBsAg, a C‐tag and a stop codon. The DNA coding sequence for the CSP‐HBsAg fusion protein was synthesized by GeneArt GmbH (Regensburg) using codons most often utilized by *P. pastoris* to encode the sequence. The insert was ligated into the expression vector pPink‐HC (Invitrogen) to construct the plasmid pPink‐HC‐R21 (Collins et al., 2017). The C‐tag was inserted by overhang polymerase chain reaction (PCR; see Supporting Information: Material 2). The PCR product and pPink‐HC‐R21 were digested with AgeI and KpnI and ligated into the pPink‐HC‐R21 backbone to make pPink‐HC‐R21‐EPEA (see Supporting Information: Material 3). Sequence identity was confirmed by sequencing of the insert.

#### 2.2.1 Generation of the recombinant *Pichia pink*

The plasmid pPink‐HC‐R21‐EPEA was linearized using the restriction endonuclease EcoN1, which cleaves once in the TRP2 locus. The linearized plasmid was then transformed into *Pichia pink* strain 1 by electroporation. The transformed yeast was grown on PAD medium (Invitrogen, A11156) and positive clones were selected for R21c expression. Full details of the process can be found in the *Pichia pink* Expression Systems manual (Invitrogen).

#### 2.2.2 Generation of R21c *P. pastoris* clone

The R21c *P. pastoris* strain (*Pichia pink* S1 R21 EPEA, 250 µl) was taken through three rounds of cell cloning at the Clinical BioManufacturing Facility (CBF), University of Oxford. The glycerol culture was thawed and streaked onto two YPDA (1% Yeast extract, 2% Peptone, and 2% bacteriological agar) plates using a sterile loop. The plates were incubated at 29°C until colonies appeared. Individual colonies were selected and after two rounds of restreaking, a total of five colonies (1.1.1, 1.2.1, 2.1.1, 2.2.1, and 2.3.1) were used to make a starter culture. Each colony was inoculated into a 50 ml tube containing 10 ml of BMGY and incubated at 29°C, 120 rpm. The increase in cell biomass was monitored by measuring the optical density of the culture at 600 nm (OD_600_) using a spectrophotometer. When the OD_600_ of the starter cultures was between 3 and 5 units (~27 h), each starter culture was diluted to an OD_600_ of 0.02 (~7 ml) in 200 ml of BMGY in a 1000 ml baffled flask. The cultures were induced 24 h postexpansion (OD_600_ 10–15 units). A sample was taken every 24 h thereafter to monitor the growth of the culture at 600 nm. For induction, the cultures were centrifuged at 500*g* for 10 min, room temperature (RT), and the pellets were resuspended in 200 ml of BMMY. About 50 ml of culture was removed from the flask 24 h postinduction to assess expression. Each culture was centrifuged at 500*g* for 10 min, at room temperature. The OD_600_ at the time of harvest was between 30 and 50 units. The pellets were re‐suspended in five times their weight in PLB. About 10 ml of the suspension was lysed at 40 kpsi using a high‐pressure cell disruption system. The lysate was incubated with 1 mM PMSF and 5 mM of MgCl_2_ at room temperature for 15 min, and then treated with 650U Benzonase®/ml followed by incubation on a rocker for 30 min. The lysate was then spun at 4700*g* for 10 min, at room temperature and the supernatant was filtered through 0.22 µm Millex HV filter. Filtered supernatant was loaded onto the 5 ml C‐tag affinity column equilibrated with three column volumes (CV) of 1× phosphate‐buffered saline (PBS), pH 7, at 2 ml/min using AKTA Prime Plus. Elution with 20 mM Tris, 2 M MgCl_2_, pH 7 produced one peak for each clone.

#### 2.2.3 Culturing *P. pastoris* clone for MCB production and expansion

The R21c clone 2.1.1 glycerol stock was thawed and 100 µl of the stock was inoculated in a sterile 15 ml tube containing 10 ml YPD. The culture was incubated at 29°C, 120 rpm and further expanded into a 3000 ml baffled flask containing 550 ml of YPD. One milliliter of culture at 2.38 × 10^9^ cells was transferred to sterile 2 ml cryogenic vials and designated as MCB before freezing at −80°C.

#### 2.2.4 Culture expansion and induction

An R21c *P. pastoris* starter culture was initiated by inoculating 20 µl of clone 2.1.1 MCB in 50 ml of BMGY in a 250 ml baffled flask. The culture was grown at 29°C at 120 rpm in a shaker incubator. At an OD_600_ of 10–20 units, 5 ml of the starter culture was used to inoculate 600 ml of BMGY in a 3000 ml baffled flask. To begin induction (at OD_600_ of 25–35 units (~24 h), the culture was spun down and the pellet washed by re‐suspending in sterile 1× PBS (pH 7.2) at 500*g*, 20°C for 10 min. The supernatant was discarded, and pellet was resuspended in BMMY and transferred to a fresh 3000 ml baffled flask. The final volume was made up to 600 ml per flask. The cultures were fed with 48 ml of 50% methanol (final concentration: 4%) 24 h postinduction.

#### 2.2.5 Harvest and cell lysis

Each 3000 ml flask was harvested 46–48 h postinduction (OD_600_ of 40–50 units), into 2 × 500 ml centrifuge tubes and centrifuged at 500*g*, 20°C for 10 min. The supernatant was removed, and pellets (17–18 g per tube) were frozen at −80°C. On the day of processing, frozen cell pellets were thawed at 18–22°C. The thawed pellets were resuspended in five times their weight in PLB and pooled in a sterile 1000 ml corning bottle. Lysis was performed by single pass of the suspension through a high‐pressure cell disruption system at 40 kpsi, 4–6°C. The protein–lipid particles assembled on lysis to form VLPs (Collins et al., 2017). The lysate was centrifuged at 4700*g*, at 4°C for 10 min to remove large debris. The autoclaved C0HC depth filter was flushed with 2000 ml of water for irrigation (WFIg) and equilibrated with 700 ml of PLB at the flow rate of 270 ml/min using a milliflex pump. Lysate was clarified at a flow rate of 70 ml/min. Both of these steps were performed at room temperature (18–22°C). The void volume of 250 ml was collected separately before starting flow‐through collection in the form of 100 ml fractions in 150 ml corning bottles.

#### 2.2.6 Removal of low molecular weight contaminants and R21c monomers

The Capto Core 700 chromatography column was equilibrated with 3 CV of PLB at 20 ml/min on AKTA Purifier 100 system. The pooled C0HC filtrate (900 ml) was loaded onto the column at a flow rate of 20 ml/min. The flow through was collected in the form of 22 × 45 ml fractions. The fractions were pooled and stored at +4°C.

#### 2.2.7 Selective binding and elution of R21c VLPs

The C‐tag affinity chromatography column was equilibrated with 3 CV of affinity equilibration buffer (20 mM Tris, pH 7). The pooled flow through from the Capto Core 700 step was brought to room temperature and loaded onto the C‐tag column at 10 ml/min. The column was then washed with 9 CV of loading buffer. The major contaminant AOX 1 was eluted from the column by washing the column with ≥12 CV of Elution buffer 1, followed by ≥12 CV of Elution buffer 2 to elute R21c VLPs from the column at 10 ml/min. Eluted VLPs were collected in the form of 1.5 ml fractions and pooled for buffer exchange.

#### 2.2.8 Buffer exchange

The pooled fractions collected from the C‐tag affinity step were dialyzed into ≥2 L formulation buffer using 30 ml slide‐a‐lyzer cassettes in pre‐autoclaved low endotoxin 4 L bottles for ~30 min at 2–8°C. This was followed by one change of formulation buffer for 30 min, and a final change for 12–18 h at 2–8°C. The dialyzed material was frozen at −80°C.

#### 2.2.9 Filtration and filling

Frozen material was thawed at 10–20°C and filtered through 0.45 µm PVDF filter and then through 0.22 µm PVDF filter in an isolator and filled into glass vials. The labeled vials were stored at −80°C.

#### 2.2.10 Analysis

Product concentration was determined by A_280_ on spectrophotometer M550 (Spectronic Camspec Limited) and calculated using the formula: (A_280nm_/extinction coefficient of 2.0) × Dilution factor. Native PAGE was used to determine the purity of high molecular weight R21c VLPs, using 7.5% Tris‐HCl gels (Bio‐Rad Laboratories Inc.). The sample was mixed (1:1 ratio) with Native sample buffer (Bio‐Rad Laboratories Inc.) and run alongside Precision Plus Protein standards (Bio‐Rad Laboratories Inc.). For Sodium dodecyl sulfate‐polyacrylamide gel electrophoresis (SDS‐PAGE), the sample was mixed with the Laemmeli sample buffer (Bio‐Rad Laboratories Inc.) in a 2:1 ratio and heated to 100°C and run on a 10% Mini Protean TGX gel (Bio‐Rad Laboratories Inc.) along with Precision Plus Protein standards (Bio‐Rad Laboratories Inc.). The gels were stained with InstantBlue™ Coomassie stain (Expedeon Limited) and the image was captured with the EZ Gel Imager (Bio‐Rad Laboratories Inc.). Western blot analysis was performed using the Trans‐Blot® turbo transfer system (Bio‐Rad Laboratories Inc.). The PVDF transfer membrane was blocked in blocking buffer (PBS/3% [w/v] Bovine serum albumin) for 1 h on a rocking platform. The transfer membrane was incubated in 1:250 dilution of mouse anti‐HBsAg immunoglobulin G (IgG) monoclonal antibody MCA4658 (Bio‐Rad Laboratories Inc.) or a 1:20,000 dilution of anti‐NANP specific monoclonal antibody for 1 h on a gyro rocker. The membrane was washed with 1× PBS and incubated in a 1:3000 dilution of secondary antibody (Alkaline phosphatase‐conjugated AffiniPure Donkey Anti‐mouse IgG (Jackson ImmunoResearch Laboratories Inc.) for 1 h at room temperature. The membrane was washed with 1× PBS and developed with BCIP/NBT (Sigma‐Aldrich) until bands appeared. The membrane was then washed with WFIg. The approximate size of VLPs was determined by electron microscopy. The sample was treated with 2% uranyl acetate before microscopy using an FEI Tecnai 12 TEM using a Gatan OneView CMOS camera. The CaptureSelect™ C‐tagXL Ligand Leakage ELISA Kit (Thermo Fisher Scientific) was used to detect residual C‐tag ligand, which can leach from the chromatography support and coelute with the protein, providing sensitivity to detect the affinity ligand contamination to less than 1 ng/ml. Residual host cell proteins (HCP) were determined using the *P. pastoris* HCP ELISA Kit (Cygnus Technologies). The HCP content of the test sample was calculated from the standard curve. In‐process host cell DNA removal was analyzed using the Quant‐iT™ PicoGreen™ dsDNA Assay Kit (Thermo Fisher Scientific) and the manufacturer's instructions on a process development run to determine C0HC step efficiency. Residual host cell DNA in the clinical batch was determined using PCR. A target‐specific qPCR assay was used to quantitate the amount of residual host cell DNA in the test article. This test was carried out externally at BioReliance (Glasgow).

#### 2.2.11 Preclinical immunogenicity study

To assess the vaccine immunogenicity, serum samples collected at Days 46 and 64 of the recovery phase from BALB/c mice administered with R21c alone or R21c mixed with Matrix M were tested for anti‐NANP IgG responses using enzyme‐linked immunosorbent assay (ELISA; antibodies to CS antigen). Total IgG ELISAs were performed in Nunc‐Immuno Maxisorp 96 well plates (Thermo Scientific) coated with 2 μg/ml NANP6C peptide in carbonate‐bicarbonate coating buffer (Sigma‐Aldrich) overnight at 4°C. Plates were washed with PBS‐Tween and blocked with 10% Casein Block (Thermo Fisher Scientific). Sera were diluted at a starting concentration of 1:3000, added in duplicate, and serially diluted threefold. Plates were incubated for 2 h at room temperature and then washed as before. Goat anti‐mouse whole IgG conjugated to alkaline phosphatase (Sigma‐Aldrich) was added for 1 h at room temperature. Following a final wash, plates were developed by adding p‐Nitrophenylphosphate (PNPP) at 1 mg/ml in diethanolamine buffer (Pierce) and optical density (OD) was read at 405 nm. Serum antibody endpoint titers were taken as the *x*‐axis intercept of the dilution curve at an absorbance value of 0.15. A monoclonal antibody against CSP was included in each assay as a reference control. Data were analyzed using GraphPad Prism version 6.03 (Graphpad Inc.), to determine the normality of distribution with the D'Agostino & Pearson omnibus normality test and for differences in medians between groups using the nonparametric, two‐tailed Mann–Whitney test.

#### 2.2.12 Stability studies

The stability of the R21c clinical batch was assessed at two different temperatures: −80°C and +2–8°C. Electron microscopy was performed to confirm the presence of particles.

## 3 RESULTS

### 3.1 Selection of R21c *P. pastoris* clone

**Figure 2:**
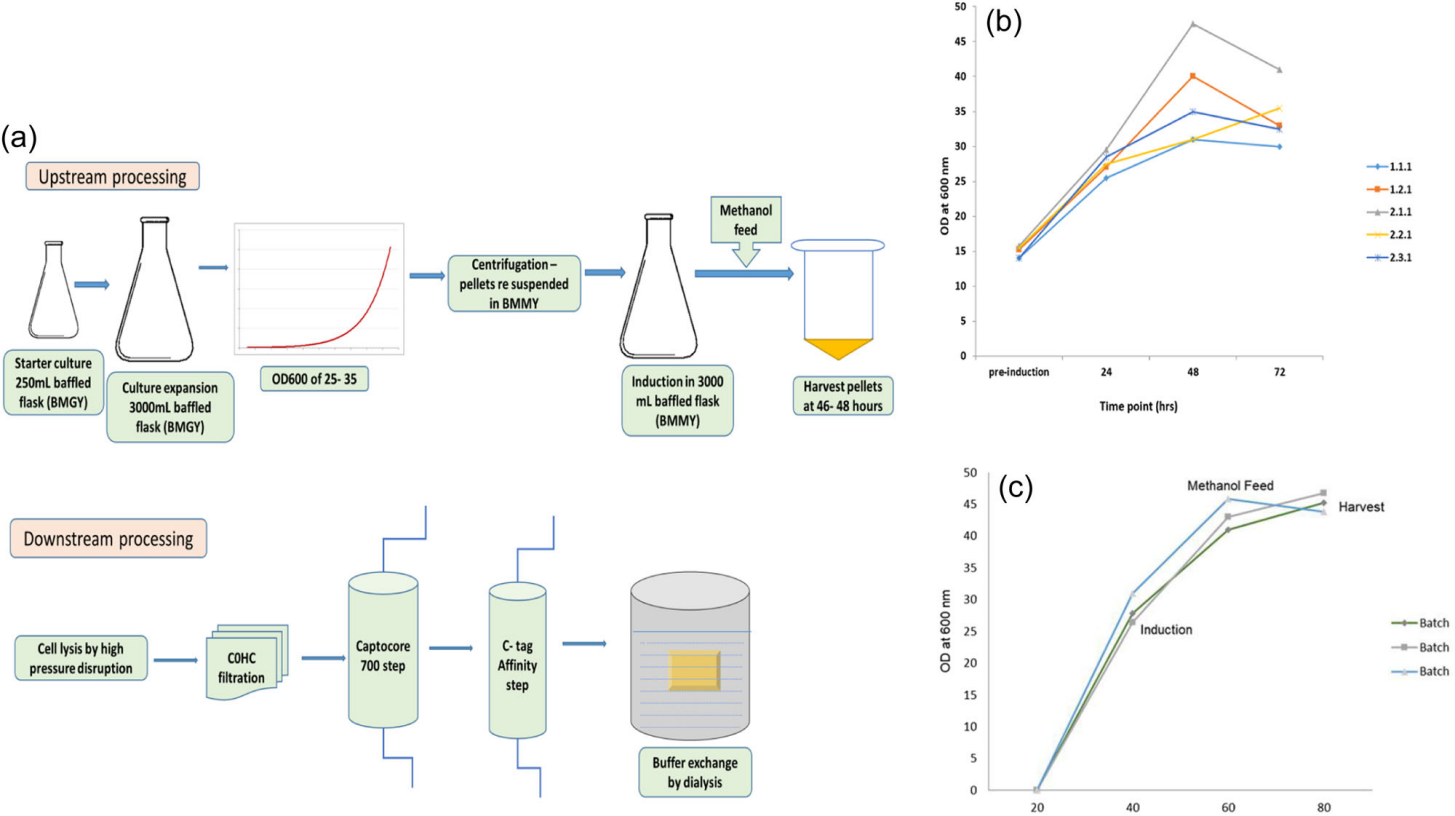
(a) Overview of the R21c production process. Upstream processing was initiated as a *Pichia pastoris* starter culture in BMGY media and expanded to 3000 ml culture. At an OD_600_ of 25–35, the cells were resuspended in BMMY, to initiate the induction under methanol. Twenty‐four hours postinduction, the cultures were supplemented with 4% methanol. Cultures were harvested 46–48 h postinduction and centrifuged. The pellets were frozen at −80°C. Downstream processing involved thawing of pellets and performing lysis using a high‐pressure disruption system at 40 kpsi. The lysate was filtered through a C0HC depth filter and the filtrate was loaded on to a Capto Core 700 column. The flow through from the Capto Core 700 column was loaded onto a C‐tag affinity column and R21c VLPs were eluted using 2 M MgCl_2_. The eluted VLPs were buffer exchanged by dialysis and frozen at −80°C. (b) Growth curves for the R21c clones. All five clones were grown in BMGY and monitored by measuring OD600 values for 72 h. The cultures were induced at an OD_600_ of 10–15 units in BMMY. (c) R21c clone 2.1.1 growth curve for three production batches. The large culture was initiated at an OD_600_ of ~0.01 units in a 3000 ml baffled shaker flask in BMGY medium. The culture was induced when the OD_600_ reached 25–35 units in BMMY medium. The culture was fed 50% methanol to a final concentration of 4%, 24 h postinduction. The cultures were harvested 48 h postinduction. VLP, virus‐like particle.

All five colonies were grown and processed for purification as described above. The presence of a peak at 280 nm positively confirmed expression of R21c for all five clones. A sample of the peak was processed for electron microscopy to confirm the presence of VLPs (Figure 4b1). Clone 2.1.1 was chosen for the production of the MCB due to the higher OD_600_ in comparison to other clones (~50 which was ~10 units higher than the next best clone 1.2.1), the presence of a peak at 280 nm post purification, and the confirmed presence of particles on electron microscopy. The consistency of growth of the clone during the production of three clinical batches is shown in Figure 2c. The small level of variance between the batches for the same clone can be attributed to the use of shaker flask where measurement of all growth parameters is limited.

**Figure 3:**
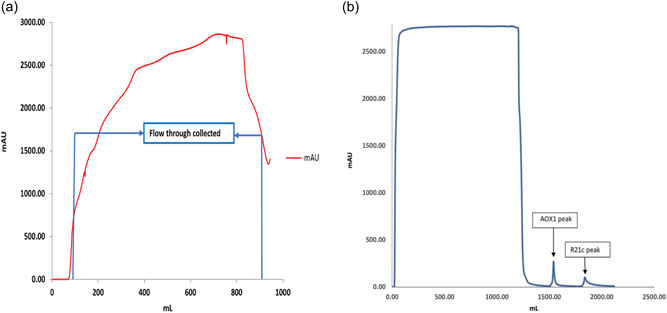
(a) Capto Core 700 chromatography. The Capto Core 700 column was equilibrated with PLB, then the clarified filtrate from the C0HC step was loaded at 20 ml/min. The flow through was collected in the form of 45 ml fractions. (b) C‐tag affinity chromatography. The C‐tag column was equilibrated with 20 mM Tris, pH 7 and the pooled flow through from the Capto Core 700 step was loaded at 10 ml/min. The major contaminant AOX 1 was eluted from the column with >12 CV of 0.1 M Elution buffer, followed by >12 CV of 2 M Elution buffer to elute R21c VLPs. Eluted VLPs were collected in the form of 1.5 ml fractions and pooled for buffer exchange. PLB, Pichia lysis buffer; VLP, virus‐like particle.

**Figure 4:**
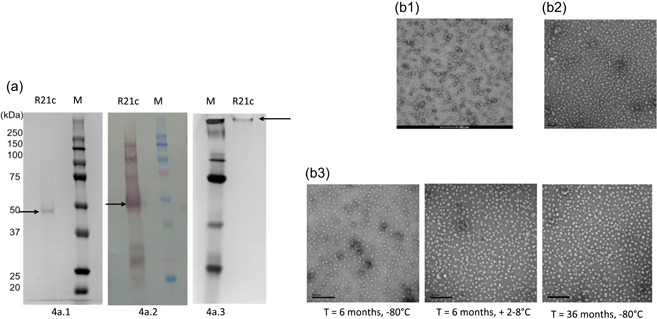
(a) Purity analysis. (a1) SDS‐PAGE: the band to the right of the molecular weight marker (M) correspond to the R21c monomer. A single band was observed on staining with InstantBlue™ Coomassie stain. Purity by SDS‐PAGE was 98.4% pure. (a2) Western blot analysis: the lane to the right of the molecular weight markers (M) shows banding pattern after staining with mouse anti‐HBsAg antibodies. The band near the 50 kDa marker is the R21c monomer. Other bands above and below the monomer band are high molecular weight structures such as dimers and proteins from HBsAg, respectively. (a3) Native PAGE: the high molecular weight band to the left of the molecular weight markers (M) is the R21c band, after staining with InstantBlue™ Coomassie. (b) Electron microscopy images of R21c particles. (b1) VLPs from Clone 2.1.1 postexpression check. (b2) 0.22 µm filtered R21c clinical product and (b3) R21c particles confirmed present at −80°C and +2–8°C up to 6 months and −80°C only at 36 months, negatively stained with 2% uranyl acetate (~20 nm). Scale indicated: 100 nm. HBsAg, hepatitis B surface antigen; SDS‐PAGE, Sodium dodecyl‐sulfate polyacrylamide gel electrophoresis.

### 3.2 Removal of host cell proteins and DNA

A total of 11 C0HC fractions were collected (F1–F11), of which fractions F2–F10 were pooled giving a total volume of 900 ml. The decision on which fractions to pool was based on previous protein concentrations performed on each fraction during the process development stage. During process development, the concentration of host cell DNA in the lysate was found to be 795 µg/ml, which reduced to 47.5 µg/ml of pool post C0HC filtration. The clinical batch sample was analyzed by an external testing organization using qPCR and residual host cell DNA was found to be 33.75 pg/ml. Residual HCP was found to be 1.64 µg/ml. All the analysis values were within the specifications set for the release of the clinical product.

### 3.3 Removal of low molecular weight contaminants and R21c monomers

The Capto Core 700 flow‐through (Figure 3a) fractions were analyzed during the process development run to confirm the presence of R21 (data not shown). This data was used to determine the fractions to be pooled for the clinical batch.

### 3.4 Selective binding and elution of R21c VLPs

The R21c VLPs were collected as either the “peak” (Figure 3b) fractions or the “tail” fractions, differing in protein concentration. The “tail” fraction was collected separately to ensure that the product would meet the specification for concentration prefiltration. Four production lots were pooled to produce one batch of R21 vaccine. The protein concentrations for the “peak” fractions ranged from 0.17 to 0.3 mg/ml and the “tail” fractions ranged from 0.07 to 0.116 mg/ml.

### 3.5 Final product yield and purity

The R21c vaccine was filled into ~400 vials at a concentration of 0.126 mg/ml in 0.3 ml volume per vial. The purity of the filled vaccine based on SDS‐PAGE analysis (Figure 4a1) was greater than 98% and the total yield was ~15 mg.

### 3.6 Residual C‐ tag ligand assay and C‐tag immune response

The concentration of Residual CaptureSelect C‐tag Affinity ligand in the clinical batch was 30.12 ng/ml (set specification <36.22 ng/ml). No significant IgG response was measured to C‐tag in mice using an experimental R21c batch (data not shown).

### 3.7 Preclinical immunogenicity

**Figure 5:**
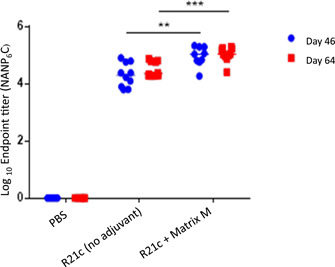
Immunogenicity of R21c in BALB/c mice. Comparison of total NANP‐specific IgG between mice injected with PBS (D46 [*n* = 5] and D64 [*n* = 6]), R21 (D46 [*n* = 9] and D64 [*n* = 10]) and R21 + Matrix M (D46 [*n* = 10] and D64 [*n* = 10]) as measured by ELISA. Bars represent median values. **p = 0.0023, ***p = 0.0001, two‐tailed). Anti‐NANP antibody titers were significantly higher in the vaccinated groups than the unvaccinated controls on Days 46 and 64. Matrix M significantly increased the response to R21c. Anti‐ NANP responses were maintained until the sampling endpoint, that is, Day 64. ELISA, enzyme‐linked immunosorbent assay; IgG, immunoglobulin G; PBS, phosphate‐buffered saline.

Anti‐NANP antibody titers were significantly higher in the vaccinated BALB/c mice than in the unvaccinated controls, and Matrix M significantly increased the response to R21c, the use of which also translates to efficacy (Collins et al., 2017) and hence considered essential for vaccine administration. Naïve sera gave zero measurable response. Anti‐NANP responses were maintained until the sampling endpoint, that is, Day 64 (Figure 5).

### 3.8 Stability

R21c particles were confirmed present at both the temperatures −80°C and +2–8°C till 6 months and −80°C only at 36 months (Figure 4b3). During all mentioned time points, quality checks like pH, protein concentration, protein degradation by SDS‐PAGE, identity by western blot analysis, and purity assessment of native gel were performed (data not shown) along with electron microscopy and found within the specifications set during product release for clinical trials.

## 4 DISCUSSION

This study illustrates the potential utility of C‐tag‐based immunoaffinity chromatography to allow GMP production of VLP vaccines in addition to soluble protein‐based candidates (Jin et al., 2018) for early phase clinical trials when the requirements of material are lower. C‐tag provided an excellent platform to produce R21c vaccine with the desired purity, yield and in the short timescale to release the vaccine for the Phase I trials without the need for exploring alternative upstream and downstream process development options.

Use of the C‐tag platform enabled the selective binding and elution of VLPs, after the removal of the monomers during the Capto Core 700 chromatography step. The C‐tag platform provides multiple advantages over conventional affinity platforms. Protein A affinity chromatography has been widely used but suffers from limitations of ligand leaching and caustic instability (Liu et al., 2009). The C‐tag antibodies linked to the resin are camelid antibodies which are only 13 kDa in size, in comparison to monoclonal antibodies which are more than 100 kDa, allowing greater density per bead and purification of larger volumes. C‐tag only consists of four amino acids, eliminating the possibility to alter protein function which can occur when larger tags like GST (glutathione S‐transferase) or MBP (maltose‐binding protein) are used. The use of polyhistidine tags often leads to significant background binding to immobilized ions when cells contain a higher percentage of histidine residues in their proteins (Kimple et al., 2013).

Significant process development was required to fulfill cGMP guidelines, but this led to the production of R21c with more than 98% purity. The clinical batch passed all the QC tests as set in the product specification. However, some rate‐limiting steps remain in the production process, which should be taken into consideration if a higher concentration and quantity of R21c is required for future, late‐phase clinical trials. First, the contaminant AOX 1 is coexpressed in high concentrations in comparison to the desired R21c. Second, there was a limited availability of binding sites for the R21c VLPs on the C‐tag resin as the pore size of the resin beads was smaller than the VLPs, thus providing only the outer surface for binding. However, an improved version of the C‐tag resin (CaptureSelect™ C‐tagXL Affinity Matrix) (unavailable during the production of this vaccine) has now been developed with larger beads (65 µm), offering an increased binding capacity compared with the previous version. Overall, the method that we have developed may not be a scalable process, but it does offer an opportunity to quickly obtain a sufficient quantity of high purity material from simple upstream systems like shaker flasks, suitable for early phase clinical trials, before investing heavily in a large‐scale cGMP purification process. Our findings support the future use of C‐tag platform technologies to enable cGMP‐compliant biomanufacturing of high purity yeast‐expressed VLP‐based vaccines for early phase clinical trials. To our knowledge, this is the first use of C‐tag technology to purify a VLP vaccine candidate for use in human clinical trials.

The R21 vaccine has now been further developed by Oxford University in partnership with the Serum Institute of India, using now a C‐tag‐free construct in matrix‐M™ adjuvant and this product has finished Phases I and IIa development. For licensed products, from the regulatory point of view, there is still a preference to have no tags and the process was hence changed by SII before they moved to phase III development. Moreover, a recently reported Phase IIb trial in Burkinabe children has demonstrated an efficacy of 77% over 1 year (Datoo et al., 2021), and a fully enrolled Phase III licensure trial in African children is in progress in four countries.

## CONFLICTS OF INTEREST

SJD is a named inventor on patents relating to malaria vaccines; AVSH and SCG are named inventors on a patent relating to R21. FJD is an employee of Thermo Fisher Scientific who is the commercial provider of CaptureSelect™ C‐tag products.

## Supporting information

Supporting information.

## Data Availability

The data that support the findings of this study are available on request from the corresponding author.
