# Supplementary material for: Production of a high purity, C‐tagged hepatitis B surface antigen fusion protein VLP vaccine for malaria expressed in Pichia pastoris under cGMP conditions
Source: Biotechnol Bioeng. 2022 Jul 22;119(10):2784–93. doi: 10.1002/bit.28181 (PMC9546177; doi:10.1002/bit.28181)
Supplement: Supplementary file 1 — Supporting information. [file BIT-119-2784-s001.docx]

Supporting information

1. Nucleotide sequence of the R21c protein

ATGGACCCAAATGCTAATCCAAATGCAAACCCTAATGCTAACCCAAACGCCAATCCAAACGCTAATCCTAATGCCAATCCTAACGCAAACCCAAACGCAAACCCTAACGCAAATCCAAATGCTAACCCAAACGCTAACCCAAATGCCAACCCAAATGCAAATCCAAACGCCAACCCTAATGCAAACCCTAACGCTAATCCTAACGCAAACCCTAATGCCAATCCAAACAAGAACAATCAAGGTAATGGTCAAGGTCACAACATGCCAAATGATCCAAATAGAAACGTTGACGAAAACGCTAATGCAAACTCTGCTGTTAAGAACAACAACAACGAAGAACCATCCGACAAGCACATCAAAGAATACTTGAACAAGATCCAAAACTCCTTGTCTACTGAATGGTCCCCATGTTCTGTTACTTGTGGTAATGGTATCCAAGTCAGAATCAAACCAGGTTCTGCTAACAAGCCAAAGGATGAATTGGATTACGCCAACGATATCGAAAAAAAGATCTGCAAGATGGAAAAGTGTTCCTCTGTTCCAGTTACCAATATGGAAAACATTACCTCTGGTTTCTTGGGTCCATTATTGGTTTTACAAGCCGGATTCTTCTTGTTGACCAGAATTTTGACCATCCCACAATCTTTGGATTCTTGGTGGACTTCTTTGAATTTCTTGGGTGGTTCTCCAGTTTGTTTGGGTCAAAATTCACAATCTCCAACCTCTAATCATTCCCCAACATCTTGTCCACCAATTTGTCCAGGTTATAGATGGATGTGCTTGAGAAGATTCATCATTTTCTTGTTCATCTTGTTGTTGTGCTTGATCTTCTTGTTGGTTTTGTTGGACTACCAAGGTATGTTGCCAGTTTGTCCATTGATTCCAGGTTCTACTACTACTAATACCGGTCCATGTAAGACTTGTACTACTCCAGCTCAAGGTAATTCTATGTTCCCATCATGTTGTTGTACCAAACCTACTGATGGTAACTGTACCTGTATTCCAATTCCATCTTCTTGGGCTTTCGCTAAATATTTGTGGGAATGGGCTTCTGTTAGATTCTCTTGGTTGTCTTTGTTGGTTCCATTCGTTCAATGGTTCGTTGGTTTGTCTCCAACTGTTTGGTTGTCTGCTATTTGGATGATGTGGTATTGGGGTCCATCCTTGTATTCTATCGTTTCTCCATTCATCCCTTTGTTGCCAATTTTCTTTTGCTTGTGGGTTTACATTGAACCAGAAGCT

1. Primers used for C- tag insertion

Forward: CCAGTTTGTCCATTGATTCCAG

Reverse: GCTCGAGGTACCTTAAGCTTCTGGTTCAATGTAAACCCACAAGCAAAAG

1. Plasmid map

The R21c protein has been placed under the control of the AOX1 promoter. The remainder of the plasmid consists of an ampicillin resistance gene and pUC replication origin to allow the plasmid to replicate in and be produced from *E. coli*, a TRP2 gene to allow insertion into the *Pichia* genome, and an ADE2 gene to allow selection of positive yeast clones.
